# Patient Recommendations to Improve the Implementation of and Engagement With Portals in Acute Care: Hospital-Based Qualitative Study

**DOI:** 10.2196/13337

**Published:** 2020-01-14

**Authors:** S Ryan Greysen, Yimdriuska Magan, Jamie Rosenthal, Ronald Jacolbia, Andrew D Auerbach, James D Harrison

**Affiliations:** 1 Section of Hospital Medicine University of Pennsylvania Philadelphia, PA United States; 2 School of Medicine University of California, Davis Davis, CA United States; 3 School of Medicine Boston University Boston, MA United States; 4 School of Nursing University of California, San Francisco San Francisco, CA United States; 5 Division of Hospital Medicine University of California, San Francisco San Francisco, CA United States

**Keywords:** patient portals, hospitalization, patient engagement, qualitative research

## Abstract

**Background:**

The inclusion of patient portals into electronic health records in the inpatient setting lags behind progress in the outpatient setting.

**Objective:**

The aim of this study was to understand patient perceptions of using a portal during an episode of acute care and explore patient-perceived barriers and facilitators to portal use during hospitalization.

**Methods:**

We utilized a mixed methods approach to explore patient experiences in using the portal during hospitalization. All patients received a tablet with a brief tutorial, pre- and postuse surveys, and completed in-person semistructured interviews. Qualitative data were coded using thematic analysis to iteratively develop 18 codes that were integrated into 3 themes framed as patient recommendations to hospitals to improve engagement with the portal during acute care. Themes from these qualitative data guided our approach to the analysis of quantitative data.

**Results:**

We enrolled 97 participants: 53 (53/97, 55%) women, 44 (44/97, 45%) nonwhite with an average age of 48 years (19-81 years), and the average length of hospitalization was 6.4 days. A total of 47 participants (47/97, 48%) had an active portal account, 59 participants (59/97, 61%) owned a smartphone, and 79 participants (79/97, 81%) accessed the internet daily. In total, 3 overarching themes emerged from the qualitative analysis of interviews with these patients during their hospital stay: (1) hospitals should provide both access to a device and bring-your-own-device platform to access the portal; (2) hospitals should provide an orientation both on how to use the device and how to use the portal; and (3) hospitals should ensure portal content is up to date and easy to understand.

**Conclusions:**

Patients independently and consistently identified basic needs for device and portal access, education, and usability. Hospitals should prioritize these areas to enable successful implementation of inpatient portals to promote greater patient engagement during acute care.

**Trial Registration:**

ClinicalTrials.gov NCT00102401; https://clinicaltrials.gov/ct2/show/NCT01970852

## Introduction

### Background

Ten years after the passage of the Health Information Technology for Economic Clinical Health in 2008 and the resulting Meaningful Use Incentive Program, most hospitals have adopted electronic health records (EHRs) [1]. The inclusion of patient portals into EHRs has been slower, but it has also been steadily rising, and it is projected to expand rapidly in the near future. According to Health Information National Trends Survey data, 5% of patients were using portals in 2008 and 17% of patients were using portals by 2013, with projections that adoption will likely increase to at least 40% by 2020 but that it may be as high as 75% [2]. In addition, there is growing evidence that portal use can improve outcomes, including medication adherence and diseases management [3], and increased patient empowerment and satisfaction with health services [4]. These trends have added urgency to the national effort to increase the use and effectiveness of portals in all phases of care; however, to date, most of this has focused on portal use in the outpatient setting [5-8].

Although the literature exploring barriers and facilitators to portal use in the outpatient setting is robust—and many demographic trends, such as age, race or ethnicity, and socioeconomic status, certainly apply across care settings [9-12]—there are also unique challenges to portal engagement in the hospital, which merit specific attention. Accordingly, there has been increased exploration of technology use in the hospital to engage patients in care in the last 5 years. A systematic review of this literature performed in 2013 describes a wide range of technologies from video games to interactive displays, mobile phone messaging, and other multimedia approaches; however, there were no studies of portal use in the hospital [13]. Our prior work explored the use of tablets [14] or smartphones [15] in a limited number of hospitalized patients, but it did not explore portal-specific barriers or facilitators to engagement. A more recent systematic review found 17 studies specifically focused on portal use in the hospital, but these studies largely explored design features and institution-specific prototypes [16]. Many of these used qualitative methods with relatively small samples (n=8-21) proportionate to the task of improving design and function. These studies have been fundamental for developing consensus around key features that inpatient portals should include [17,18]; however, as more hospitals are now adapting existing platforms rather than designing their own, a deeper understanding of patient experiences using existing portal platforms from a large and diverse inpatient sample is needed to speed up the process of successful implementation in acute care.

### Objectives

Accordingly, we conducted in-person, bedside interviews with 97 patients who were provided with a tablet and access to a widely used institutional portal (Epic MyChart). Our objectives were to obtain a deeper understanding of patent perceptions of using a portal during an acute episode of care and explore patient-perceived barriers and facilitators to portal use during hospitalization. On the basis of our findings, we developed specific recommendations for hospitals and health systems on how to improve portal implementation to maximize patient engagement.

## Methods

### Study Design, Participant Enrollment, and Portal Characteristics

This study reported the qualitative data collected from debrief interviews with adult patients hospitalized for a general medical condition at the University of California, San Francisco (UCSF), who participated in a randomized controlled trial (RCT) of a focused educational intervention to increase engagement with the portal (ClinicalTrial.gov identifier NCT02109601). Eligibility criteria included the following: admission to the general medicine service, age 18 years or older, and the ability to communicate in English. Exclusion criteria included the following: admission to the intensive care unit (ICU), cognitive impairment, or isolation precautions. Quantitative outcomes data of the randomized trial are reported in a separate paper [19]; here, we discuss qualitative results from debrief interviews with all patients who completed the trial.

Research assistants (RAs) screened patients via UCSF’s EHR (Epic) and obtained written consent from those willing to participate. If a participant did not have an active institutional portal account (UCSF MyChart), the RA assisted with registration and activation of a new account. All enrolled participants received an iPad tablet (iPad 16 GB third generation Model A1430) to use for that day only. Participants were instructed on basic iPad features, including how to use the keyboard, home button, and touch screen. As per our RCT protocol reported previously [20], intervention patients (n=50) received an in-person, bedside tutorial on how to navigate the portal, with specific focus on how to perform key tasks that included viewing their test results, viewing medications, messaging with providers, and scheduling appointments. Control patients (n=47) received assistance only with logging in to the portal as needed, but they did not receive detailed guidance or assistance on using the portal to perform the specific tasks above. The UCSF Committee on Human Research (Institutional Review Board) approved the research protocol.

The portal used in this study (Epic MyChart), as configured by UCSF, had the following characteristics. As with most portals, patients can access only certain content, not all information in EHR. UCSF’s MyChart provides patient access to many features of the EMR, which are standard in the MyChart platform, including Allergies, Demographics, Health Goals, Medications, Problems, Immunizations, Care Team, Documents, Health History, Lab Results, Plan of Care, Procedures, and Vitals. Most lab results (eg, common blood tests, such as complete blood count or comprehensive metabolic panels) are available in real time, whereas others (eg, advanced imaging results from computed tomography) are released after a 24-hour delay. More detailed information about the portal can be obtained from the Terms and Conditions published on the UCSF MyChart website [21].

### Data Collection

Interviews were conducted by 2 RAs (YM and RJ) who received study-specific training in qualitative interviewing techniques. RAs performed 1 interview with each patient at the time the study iPad was recollected, comprising 10 questions: 4 multiple choice questions about patient satisfaction and 6 open-ended questions about their experience (Multimedia Appendix 1). RAs read all interview questions aloud to patients and manually transcribed their responses into a single, secure website (REDCap) in real time [22]. RAs were trained to probe deeper into patient’s initial responses using follow-on queries, such as “Just to be sure I understand, what do you mean by X” or “That’s interesting, can you tell me more about Y?” RAs read patient responses back to the patient before finalizing each entry to confirm accuracy. To ensure high-quality data collection, 3 study investigators (JDH, ADA, and SRG) gave weekly feedback to RAs, and the first author (SRG) met with RAs daily to review content and provide assistance and guidance.

### Data Analysis

We analyzed qualitative data from open-ended questions using a thematic analysis approach [23]. A total of 2 authors (YM and JR) independently performed primary coding using all of the interview data, and they resolved any discrepancies in the individual codes through negotiation. A third author (SRG) performed secondary coding by reviewing all data and modifying the initial code sheet iteratively as needed to capture all conceptual domains observed in the data. Finally, a fourth author (JDH) reviewed the final code sheet along with data (quotes) to support each code and participated (along with the entire coding team YM, JR, and SRG) in the development of themes through integration of multiple codes into overarching concepts. All study authors reviewed and agreed on the final code structure, which contains 18 codes integrated into 3 overarching themes (Multimedia Appendix 2) framed as patient recommendations to improve portal use in the hospital. We used STATA version 13.1 (College Station) to perform frequency analysis and describe participant characteristics, including age, race, gender, electronic device ownership, frequency of devise use, and frequency of internet use.

## Results

We enrolled a diverse sample of 97 hospitalized patients (Table 1). Fifty-three (53/97, 55%) were women, and in terms of race/ethnicity, 44 (44/97, 45%) were nonwhite: 14 (14/97, 14%) black, 9 (9/97, 9%) Asian, and 21 (21/97, 22%) other/declined.

The average age was 48.1 years (range 19-81 years), and the average length of hospitalization was 6.4 days. In terms of previous use of technology, 67 participants (67/97, 69%) reported owning a laptop computer, 57 participants (57/97, 59%) owned a smartphone, 51 participants (51/97, 53%) owned a desktop computer, and 48 participants (48/97, 49%) owned a tablet computer. Only 6 participants (6/97, 6%) did not own any of these devices. In addition, 79 participants (79/97, 81%) had previously looked up health information on the Web, 55 participants (55/97, 57%) had used the internet to communicate with a health care provider, 39 participants (39/97, 41%) had scheduled a medical appointment on the Web, and 34 participants (34/97, 36%) had refilled a prescription for a medication over the internet. With regard to use of the institutional portal (UCSF MyChart) specifically, 52 patients (52/97, 54%) had previous experience (had active accounts) and 45 patients (45/97, 46%) were new users (registered for a new account as part of this study).

Overall patient-reported experience with the tablet and portal was very high: 78 patients (78/97, 80%) were satisfied or very satisfied with using the tablet in the hospital, and 83 patients (83/97, 86%) were satisfied or very satisfied using the tablet to access their portal. Qualitative analysis reinforced this, and most patients offered suggestions about how their experience could be enhanced or expanded to include other patients. We organized these suggestions into 3 overarching and integrating themes: (1) hospitals should provide access to a device and bring-your-own-device (BYOD) platform to access the portal; (2) hospitals should provide an orientation on how to use the device and the portal; and (3) information in the portal should be easy to understand and up to date.

**Table 1:** Participant characteristics (N=97).

Characteristics				Values, n (%)
**Demographics**				
	**Age (years)**			
		18-49	48 (49)	
		50-60	41 (42)	
		≥70	8 (8)	
	Female, gender			53 (55)
	**Race or ethnicity**			
		White	53 (55)	
		Black	19 (20)	
		Hispanic	9 (9)	
		Asian	7 (7)	
		Other/unknown	9 (9)	
	**Insurance**			
		Medicaid	23 (24)	
		Medicare	22 (23)	
		Private	26 (47)	
		Self-pay/uninsured	6 (6)	
**Technology use characteristics**				
	Own desktop computer			51 (53)
	Own laptop computer			67 (69)
	Own smartphone			57 (59)
	Own tablet computer			48 (49)
	Does not own a device			6 (6)
	**Internet use**			
		Daily	79 (81)	
		Several times a week	7 (7)	
		Once a week or less	6 (6)	
**Prestudy Web-based health tasks**				
	Looked up health information			78 (80)
	Communicated with provider			55 (57)
	Scheduled medical appointment			39 (41)
	Refilled prescription			34 (35)
	None of these			10 (10)

### Recommendation 1: Hospitals Should Provide Access to a Device and Bring-Your-Own-Device Platform to Access the Portal

Overall, the most consistent feedback received was that access to a device and the portal was a very positive experience, and, accordingly, many participants felt strongly that our hospital should strive for this level of engagement as standard of care. When we probed deeper, we discovered there were actually several components worth exploring separately. First, patients recommended the hospital provide devices to every patient who wanted one:

It would be nice to give loaners [iPad] out to any patient who wants one.

50-year-old woman, new portal user

All patients should get a device as opposed to waiting for patients to request one.

60-year-old woman with previous portal experience

Furthermore, patients also offered suggestions regarding the use of devices once deployed. The overall concept expressed most consistently was that hospitals should provide multiple options to increase accessibility of the portal by ensuring that the device itself (or accessories) was adaptable to needs of patients:

I wished it [the tablet screen] was not as touch sensitive because sometimes my hands shake and I end up select things without me wanting to.

38-year-old woman, new portal user

Sometimes my device won't respond because I have callouses from burns lack of circulation in my fingers and these devices work with thermal. So not sure if it is me or the device.

41-year-old man with previous portal experience

Patients also suggested ways that accessories or modifications to the device could maximize their ability to engage during their hospital stay. Some patients suggested modifications that could represent changes to both the device and some function of the portal as well (eg, voice recognition and transcription):

Provide accessibility programs [such as screen reader] and headphones for patients with poor vision.

35-year-old woman, new portal user

[It would be nice if the iPads had the ability of transcribing after you speak, such as in Google. This iPad didn't have that ability, is that Siri? Having Siri on MyChart would be very nice!

47-year-old man with previous portal experience

Finally, with regard to optimizing patient opportunities to access the portal and engage meaningfully with it during hospital care, patients also suggested the development of a BYOD approach:

The iPad is fine, but I like to use my own smartphone. It is actually both a phone and tablet. I'm more comfortable because it's my own and also because it's an android and I feel better using it, I'm more familiar with it. I just feel more comfortable with that.

41-year-old man with previous portal experience

It seems like this [project] is specific to iPad tablets, but other devices such as androids would also work and in case that patients could bring their own device.

67-year-old man with previous portal experience

### Recommendation 2: Hospitals Should Provide an Orientation on How to Use the Device and the Portal

Overall, most patients expressed high satisfaction with the orientation they received to the device and the portal. Interestingly, some patients with previous experience articulated the value of reviewing basic use and key functions of the portal to ensure familiarity and competency with these functions before addressing more advanced functions or topics, especially given changes and updates in the portal that occur over time:

The tutorial was very helpful for me because I have been trying to get signed up on this thing for a while, but each time I tried, I had issues. And it’s hard, because you know I am sick and having to deal with one more thing was just overwhelming. It was nice to have you help me through this process finally.

38-year-old woman, new portal user

I have been using [the portal] for two years, and I have seen different versions. It seems like with time, it gets more confusing. It is not as intuitive anymore, and you have to guess your way around to accomplish the same tasks.

42-year-old man with previous portal experience

In addition to general orientation (or reorientation) related to basic functions, which could be tailored to the participant’s level of previous experience, several participants suggested that special modifications be made on the basis of other characteristics, such as age and level of technological savvy or sophistication:

It would be a good idea to do a focus group with older folks to see if they like tablets, if they want to use them, if it is easy to use, etc. Some people may not want to participate because they don't have computers.

67-year-old man with previous portal experience

For someone not as techy, maybe walk patients through a tutorial for those who don't know how to use it. Maybe have a test web site or have a little Q&A.

38-year-old man, new portal user

Regardless of age, being technology savvy, or level of prior portal experience, many patients expressed a desire for assistance with device settings to optimize their experience. Often, these were very basic issues, such as how to adjust font size:

Maybe at the beginning it would be nice making the font bigger. Maybe bold letters to highlight topics such as test results, or have a button that says: 'can you see this/read this?' and picture of the magnifying glass to make it bigger for older people not as tech savvy…it was hard to read even for me and I have 20/20 vision and I had to make the page larger.

38-year-old man, new portal user

Some links in MyChart were too close together and it was hard to tap the right choice. Larger font will help to be able to see better. Also, not knowing how to use the iPads, the interface was a mystery.

48-year-old man, new portal user

Perhaps, most surprisingly, even patients with previous portal experience and those who felt confident in their ability to use the device and navigate the portal expressed a desire for more assistance with the first steps of access: remembering the Web address for the portal (URL institution-specific portal) and their log-in information (username and password):

I have been trying to get signed up on this thing [portal] for a while. My doctor has been telling me about it for a long time, but each time I tried, I had issues. Either it didn't recognize my username or password and it was just difficult.

38-year-old woman, new portal user

Sometimes confusing if you google “UCSF MyChart,” it won't take you to the MyChart page that would allow me to login and I can’t remember the right web address.

76-year-old man with previous portal experience

### Recommendation 3: Hospitals Should Ensure Portal Content is Up to Date and Easy to Understand

Participants frequently commented on the lack of timely information in their portal. Several participants suggested that the portal would be more useful to them in the hospital if it had more frequently updated information. Others suggested they would prefer to be able to see “everything” in terms of results rather than have access only to results from a limited set of labs, imaging studies, or procedures:

There wasn’t much [on the portal] but my medications at the moment. I wanted to see more information about my tests; that would be nice.

42-year-old woman, new portal user

MyChart could have more features and it could be updated quicker: I don't mind a data dump of what physicians see.

42-year-old man with previous portal experience

Many participants highlighted challenges they encountered in understanding the content in the portal in terms of medical terminology or “jargon” in their portal. Often, patients expressed a desire to increase both quantity (more data) and quality (more interpretation) of information to enhance the meaning and applicability of information to guide their hospital care:

Half of the medical information there [in the portal] is hard to understand. Maybe it’s easier for someone with a medical background.

38-year-old man, new portal user

I don't like the list of your diseases in MyChart because when you click on your disease, it gives very generic information…it's really not that helpful. I want it to be more personalized to my illness.

61-year-old woman with previous portal experience

In summary, patient feedback revolved around the experience of being hospitalized and the heightened desire for information in this setting. Accordingly, patients directed suggestions for improvement toward the hospital to increase engagement with their portal during acute care.

## Discussion

### Principal Findings

This qualitative study of patient portal use during hospitalization is one of the largest, in-depth explorations of the patient experience in a highly diverse sample of inpatients using a widely used platform (Epic MyChart). Previous studies have focused on patient suggestions for design aspects of an ideal portal or policies to promote broad adoption [13-24]; our study builds on this literature to characterize fundamental issues to implementation. Indeed, the most consistent feedback was not about developing advanced new features; patients focused on basic issues, such as providing universal access, orientation, and information. These are issues largely within the control of the hospital, but these may go overlooked, as they seem “simple” and may therefore represent underappreciated barriers to successful implementation. Indeed, many of the issues identified by patients represent foundational issues, which, if not resolved on “day one” of a patient’s hospitalization, are likely to prohibit more meaningful, longitudinal use of the portal throughout their hospital stay. There are several ways through which our findings could inform implementation and optimization of patient portals in the hospital.

First and foremost, patients in our study felt strongly that access to a device and support for a BYOD approach were fundamental to ensuring broad and meaningful engagement. Relatively few hospitals have taken the approach of placing a patient-facing device in every room; generally, this has been focused to construction of new hospitals [25,26]. Other institutions have supplied tablets to patients, as needed, in specific units, such as oncology and the Medical ICU [27]. Moreover, applying guidelines for BYOD use in the hospital [28] and providing devices just for patients who do not bring their own may suffice, given that many (if not most) now bring their own devices with them to the hospital [15]. A second step toward universal access could be broader adoption of Application Program Interfaces to integrate more seamlessly with device-specific programs, such as Apple’s new Heath Records section, which can securely and automatically interface with the EMR from 40 health systems [29]. Finally, with respect to access, it should always be recalled that even patients who bring their own device may still need additional assistance with device use and portal access in the setting of acute illness and hospitalization. Even patients who ordinarily navigate a touch screen interface may need ease-of-access assistance with a keyboard, mouse, or headphones.

Second, patients in our study felt strongly that hospitals should go beyond access to devices and the portal and ensure all patients are adequately oriented to both the device and portal to facilitate engagement. This finding is in alignment with previous studies of other stakeholders, including clinicians, information systems leaders, and administrators [17,30]; however, there are few that focus on the specific challenge of patient education to leverage technology within the hospital and even fewer that specifically focus on portals [20,31]. Indeed, a recent systematic review by Roberts et al [32] described 9 studies focused on familiarization, training, and ongoing support of technology use during hospitalization, but only 1 study focused on the EHR portal [33]. In this study, the issues identified may seem relatively simple, but they are also foundational; thus, they can represent critical barriers that should be addressed on “day one” of hospitalization. Fortunately, implementation solutions for these issues may be relatively straightforward and present hospitals with opportunities for “quick wins.” For example, offering an overview orientation to devices and portals to every patient would likely require some combination of standardized approach (eg, Web-based tutorial), as well as the ability to provide individualized assistance as needed (eg, frontline providers) [34]. Some patient groups may also need approaches specifically tailored to them, such as older [9] and even middle-aged patients [10]. Regardless of age, many patients in this study requested assistance with adjusting features of the tablet, such as font size. Even patients who owned the same tablet sometimes needed assistance to configure the device for optimal portal use, which aligns with our clinical experience with caring for the hospitalized—they are often not able to accomplish simple self-care tasks that they would otherwise do independently when they are not acutely ill. These issues may be overlooked in many hospitals because of assumptions or inferences about patient experiences and preferences, as well as patients’ own hesitancy to ask for help in these areas, if not specifically prompted or offered assistance.

Third, patients in this study recommended that hospitals maximize efforts to ensure the content of portals is up to date and easier to understand, which builds on recent studies. O’Leary et al interviewed 18 hospitalized patients, which emphasized the desire for more information and greater assistance with interpretation [30]. Dalal et al identified challenges about communicating care plans through analysis of messages sent via the portal by 158 hospitalized patients [35]. Similarly, a recent scoping review by Roberts et al found an overarching theme of interactive learning for patients, noting “patients are more accepting of, engaged in, and satisfied with education that is tailored to reflect their personal situation” [29]. Similarly, patients in this study wanted access to more information and wanted it to be delivered more quickly. Findings from this study add further support to recent studies [36,37], suggesting that the default should be to release results automatically, unless specifically requested by the ordering clinician. Although releasing more data directly to patients in a “show me everything” fashion may complicate the challenges of making information in the portal easier to understand, the development of more robust dictionaries with hover or mouse-over functionality, links to high-quality health information sites, and support for self-monitoring programs could help mitigate this problem and improve patients’ abilities to engage with their results in real time [38].

This study has several limitations. First, it is a single-site study using 1 EHR portal (Epic MyChart), which may limit generalizability to other systems; however, the Epic platform is the most widely used in the United States, which ensures broad applicability. Second, we provided only 1 device (Apple iPad); we did not provide a variety of devices for patients to choose from or study portal interactions using patient-supplied devices (BYOD). Nonetheless, the themes we present here are relevant to any device a patient might use to access the portal during hospitalization (whether hospital-provided or BYOD); thus, they have broad applicability. Third, as with any qualitative study of patient perspectives, there is potential for participant biases to effect results. To minimize the potential for recall bias, we interviewed patients on the same day, on which they were asked to access their portal; in addition, to minimize social desirability bias, we framed questions to solicit open-ended feedback and avoided close-ended questions, such as whether patients liked or disliked certain features. Fourth, we only enrolled patients who were cognitively intact and could provide feedback on their personal experience with the portal; we did not interview family members or caregivers who are especially important in the care of cognitively impaired patients. Finally, we only gave the participants the opportunity to use the iPad for 1 day, and responses might be different if they were interviewed after having more time to use the device or even after leaving the hospital. Future studies should attempt to address this limitation by following patients longitudinally, to understand how their experiences and needs may vary when transitioning from acute care to postacute care and recovery from hospitalization.

### Conclusions

In conclusion, our qualitative findings from a study of a large, diverse sample of hospitalized patients highlight opportunities to improve hospital implementation and patient engagement with the portal care in 3 key areas: access, orientation, and usability. Our findings have important implications for the successful deployment of acute-care patient portals, and they suggest several hospital-level interventions to speed implementation of existing platforms. As patients become increasingly engaged with mobile and connected devices in their personal lives, expectations for the use of these technologies to facilitate better engagement during hospitalization will continue to grow. Optimization of their experience via the patient portal is a first and critical step toward realizing the potential for these technologies to improve outcomes in inpatient care.

## Appendix

Multimedia Appendix 1 Study debrief interview.

Multimedia Appendix 2 Codes and themes.

## Abbreviations

BYOD: bring-your-own-device
EHR: electronic health record
ICU: intensive care unit
RA: research assistant
RCT: randomized controlled trial
UCSF: University of California, San Francisco
