# Supplementary material for: Patient Recommendations to Improve the Implementation of and Engagement With Portals in Acute Care: Hospital-Based Qualitative Study
Source: J Med Internet Res. 2020 Jan 14;22(1):e13337. doi: 10.2196/13337 (PMC6996719; doi:10.2196/13337)
Supplement: Multimedia Appendix 1 [file jmir_v22i1e13337_app1.docx]

Multimedia Appendix 1: Inpatient MyChart Study Debrief Interview

Multiple-choice satisfaction questions

1. Please tell us about your satisfaction with using the iPad for browsing the Internet.
2. Please tell us about your satisfaction with using the iPad for entertainment purposes.
3. Please tell us about your satisfaction with using the iPad for viewing MyChart.
4. Please tell us about your overall satisfaction using the iPad today.

Answer choices for questions 1-4 were: very satisfied, satisfied, unsatisfied, and very unsatisfied.

Open-ended experience questions

1. How would you describe your overall satisfaction with the iPad today?
2. Did you have difficulty using the iPad to do what you wanted to do?
3. Please tell me about accessing MyChart: was it useful to you?
4. What were you able to do using the MyChart today?
5. What barriers or challenges did you encounter in using MyChart?
6. How could we improve your experience using the iPad to access MyChart in the hospital?
