# Supplementary material for: Patient Recommendations to Improve the Implementation of and Engagement With Portals in Acute Care: Hospital-Based Qualitative Study
Source: J Med Internet Res. 2020 Jan 14;22(1):e13337. doi: 10.2196/13337 (PMC6996719; doi:10.2196/13337)
Supplement: Multimedia Appendix 2 [file jmir_v22i1e13337_app2.docx]

**Multimedia Appendix 2: Patient Interview Codes and Integrated Themes**

| **Codes** |
| --- |
|  |
| 1. Patient experience with tablet provided by study (iPad) |
| 1. Patient preferences for another device to be provided by hospital (e.g. laptop) |
| 1. Patient preference to specifically bring/use own device |
| 1. General comments about orientation to device and portal |
| 1. Comments specific to re-orientation (e.g. not first time user) |
| 1. Comments related to device settings (e.g. how to adjust font size) |
| 1. Comments related to login (e.g. bookmarking login page and saving login info) |
| 1. Challenges with touchscreen (including keyboard function or clicking) |
| 1. Suggestions for other modalities such as keyboard, mouse, voice, gesture recognition |
| 1. Patient preferences to have data limited/focused (avoid data overload) |
| 1. Patient preferences to have unlimited data (“show me everything”) |
| 1. Patient preferences to have most-recent results, up to the minute (avoid lag/delays) |
| 1. Patient preferences to have access to archive (results from long ago) |
| 1. Patient suggestions for assistance understanding and interpreting results |
| 1. Descriptions of portal activities accomplished (“I logged in and viewed my meds”) |
| 1. Description of activities not accomplished (“I was not able to find appointments”) |
| 1. Global impressions/assessments without specifics (“It was great” or “I didn’t like it”) |
| 1. Suggestions for messaging function (e.g. send messages to hospital MD or RN) |
|  |
| **Overarching Themes (numbers for codes integrated into each theme)** |
|  |
| 1. **Hospitals should provide a device and support BYOD to access the portal** |
| - 1. Make a device available to everyone who wants/needs one (codes 1, 2, 17) |
| - 1. Provide multiple options to increase accessibility of portal (codes 8, 9) |
| - 1. Provide a BYOD platform or mobile version of portal webpage (codes 3, 15, 16) |
|  |
| 1. **Hospitals should provide an orientation on how to use the device and the portal** |
| 1. General orientation issues for new vs. experienced users (codes 4, 5) |
| 1. Orientation issues specific to users who are older or less tech-savvy (codes 5, 6, 7) |
| 1. Assistance optimizing device for portal use (codes 4, 6, 8) |
| 1. Assistance with login and bookmarking (codes 6, 7) |
|  |
| 1. **Hospitals should ensure portal content is up-to-date and easy to understand** |
| 1. Avoid delays in releasing information (codes 10, 11, 12, 13) |
| 1. Assist with interpretation of results and medical terminology (codes 14, 16, 18) |
